# Multi-Robot Preemptive Task Scheduling with Fault Recovery: A Novel Approach to Automatic Logistics of Smart Factories

**DOI:** 10.3390/s21196536

**Published:** 2021-09-30

**Authors:** Vivian Cremer Kalempa, Luis Piardi, Marcelo Limeira, André Schneider de Oliveira

**Affiliations:** 1 Graduate Program in Electrical and Computer Engineering, Universidade Tecnológica Federal do Paraná (UTFPR), Av. Sete de Setembro, 3165, Curitiba 80230-901, PR, Brazil; piardi@ipb.pt (L.P.); limeira@alunos.utfpr.edu.br (M.L.); andreoliveira@utfpr.edu.br (A.S.d.O.); 2 Department of Information Systems, Universidade do Estado de Santa Catarina (UDESC), Luiz Fernando Hastreiter St., 180, São Bento do Sul 89283-081, SC, Brazil; 3 Research Center in Digitalization and Intelligent Robotics (CeDRI), Instituto Politécnico de Bragança (IPB), Campus de Santa Apolónia, 5300-253 Bragança, Portugal

**Keywords:** Multi-Robot Task Allocation, Multi-Robot Preemptive Task Scheduling, fault recovery, smart factories, warehouse logistics

## Abstract

This paper presents a novel approach for Multi-Robot Task Allocation (MRTA) that introduces priority policies on preemptive task scheduling and considers dependencies between tasks, and tolerates faults. The approach is referred to as Multi-Robot Preemptive Task Scheduling with Fault Recovery (MRPF). It considers the interaction between running processes and their tasks for management at each new event, prioritizing the more relevant tasks without idleness and latency. The benefit of this approach is the optimization of production in smart factories, where autonomous robots are being employed to improve efficiency and increase flexibility. The evaluation of MRPF is performed through experimentation in small-scale warehouse logistics, referred to as Augmented Reality to Enhanced Experimentation in Smart Warehouses (ARENA). An analysis of priority scheduling, task preemption, and fault recovery is presented to show the benefits of the proposed approach.

## 1. Introduction

Industrial processes are changing with the introduction of new technologies, aiming to improve productivity and create more flexible products. This modernization is not standard static automation, but advanced manufacturing through interconnected dynamic agents. Smart factories are composed of a group of agents (i.e., robots, machines, sensors) with machine-to-machine connectivity that can exchange information and make decisions without compromising production, ensuring continuous flexible manufacturing in a Cyber-Physical System (CPS) [1].

Flexible manufacturing is achieved with the introduction of dynamic agents that can adapt to new demands and requirements [2]. Robots are the most flexible agents of manufacture, directing interactions with other active agents, such as conveyors, storage, and production machines. In this context, smart factories are sharply increasing the use of mobile robots, enabling a new class of automation based on multi-robot systems.

Multi-robot systems (MRS) can be understood as the cooperation, coordination, or interaction of a group of mobile robots to achieve a single task or a distinct set of tasks [3]. A large group of mobile robots is known as a robot swarm [4]. The MRS has different definitions; for example, in [5,6] the swarm was defined as the coordination between large groups of relatively simple robots through the use of local rules. Gerardo Beni [7] stated that a group of robots has some unique characteristics, also found in swarms of insects: decentralized control, lack of synchronization, and simple and (almost) identical members. All these definitions are based on groups of primitive robots with elementary skills, precisely the opposite of mobile robots with advanced abilities used in smart factories.

MRS has been introduced in industries to improve production and increase flexibility. Thus, robots must work as operators in dynamically assigned tasks. The task is a sub-goal that is necessary to achieve the overall purpose of the system, which can be made independently of other sub-objectives [8]. However, task allocation should not only ensure that the whole mission is achieved, but also that the tasks are productively and efficiently distributed among the robots. A useful task allocation approach must consider available resources, the factors to be optimized, and the capacity of the robots [9].

Benefits of MRS employment are dependent on an efficient decomposition of production tasks because each robot or group is responsible for accomplishing a sub-task. Task allocation in MRS is not trivial, especially when considering unreliable heterogeneous robots, equipped with different resources, designed to execute several distinct tasks with various requirements and constraints [10]. Multi-Robot Task Allocation (MRTA) is widely discussed [11,12,13,14,15,16], where the challenges are the allocation of complex tasks, allocation of dynamic tasks, allocation of highly restricted tasks, and heterogeneous allocation.

In addition, other challenges need to be addressed for MRS application to be effective [17], such as

1. Big Data: it remains a challenge to use big data in MRS, which has computational and communication limitations;
2. Internet of Things (IoT): issues such as communication, consensus, information flow, and security are IoT challenges in robotics;
3. Task Complexity: in dynamic environments, it is important to allow task decomposition automatically in order to perform the re-planning as the environment changes;
4. Autonomous Machine Learning: enables MRS agents to work better in dynamic environments;
5. Scalability and Heterogeneity Tradeoff: dynamic environments make this topic a challenge;
6. Coalition Formation and Task Allocation: coalitions may have to change during the performance of tasks and need to plan some form of fault tolerance;
7. Human-in-the-Loop: it can be challenging due to additional communication overhead;
8. Transfer Learning: this topic needs to be tested on real-world systems with complex tasks;
9. Unified Framework: many works have developed modules separately and made developing an automated MRS challenging.
10. Other challenges: communication limitations, uncertain connectivity, and lack of an evaluation standard are examples of open MRS problems.

This work discusses challenges three and six. The task complexity issue is addressed through an approach that breaks down system processes into more simple tasks to the distributed execution through multiple autonomous robots. For the problem of coalition formation and task allocation, we present a new approach to subgroup formation and cooperative accomplishment, which composes the complex logistic process.

This paper presents a novel approach for MRTA that introduces priority policies on preemptive task scheduling, considering the dependencies between tasks, and tolerating faults, called MRPF. The proposed approach contains four main elements. (1) Allow processes with dependency or resource constraints, requiring more effort in coordinating robots, to achieve the objective with independent tasks. (2) Provide a method for forming subgroups (coalitions) of robots for the cooperative accomplishment of priority processes with significant resource requirements. As such, several groups of robots can perform different tasks at the same time. (3) Preemptive scheduling of tasks, allowing tasks with higher priorities to be completed as soon as they enter the task queue while delaying lower priority tasks. (4) Present a multi-robot scheduler supporting fault recovery, allowing a robot to be replaced in case of failure.

Concerning challenge six, the proposed approach has the prerogative of being a fault-resilient scheduler that ensures the correct and continuous operation in automatic logistics systems. The main purpose is to support failures so that the logistic process remains operational without requiring the classification of failures.

The rest of this paper is organized as follows. Section 2 discusses the related work. Section 3 presents the problem statement and the assumptions for MRTA. Section 4 explains the technical challenge and inspiration. Section 5 presents the proposed approach to MRPF. Section 6 discusses the experimentation and evaluation in real warehouse logistics. Section 7 presents a comparison with other approaches. Finally, Section 8 presents the conclusions.

## 2. Related Work

MRTA is complex because tasks are dynamic and continuously changing. Each task has specific requirements and distinct time constraints, and the task distribution is very heterogeneous. In attempting to solve the MRTA problem, several approaches have emerged. Primarily, these are: (1) behavioral approaches, (2) market-based approaches, and (3) optimization-based approaches.

Behavioral approaches focus on acting patterns incorporated into the agents that are activated or deactivated as a reaction, according to the stimuli received. If the behavior is enabled, the robot will perform some pre-programmed actions. Multiple behaviors may be active at the same time, so there must be rules for combination or prioritization [18]. The most prominent behavioral algorithms are: ALLIANCE [19], Broadcast of Local Eligibility (BLE) [20], and ASyMTRe-D [21]. Behavioral approaches are best suited to consider time constraints in MRTA; however, they are still only a small part of a “conceptual solution” because they are only locally optimal [22].

Market-based approaches to the MRTA problem, such as [23,24], involve explicit communications between robots. Robots bid on tasks based on their capabilities. Trading between robots is based on the market theory, where the objective is to optimize an objective function, taking into account the utility values of the robots [25]. As a disadvantage, this approach presents the possibility of overly demanding a lot of computing and communication [26]. The algorithms with the most prominence based on market laws are: M+ [27], Murdoch [28], TraderBots [29], and S+T (*Services and Tasks*) [30]. This approach, however, can fail if task allocation requires a high cost of communication between robots. Only theoretically can this approach guarantee optimal task allocation. Therefore, market-based approaches fit the allocation of small-scale and medium-scale tasks [22,31].

Optimization-based approaches are designed to find the ideal solution from a set of available solutions. Optimization methods have a set of constraints, and the ideal solution is chosen according to a certain criterion, which defines the objective function. Because bio-inspired stochastic approaches are best suited to work with distributed systems for multiple robots, many researchers have developed studies in this area, such as: Refs. [32,33,34,35], with genetic algorithms; Refs. [22,36], with ant colony optimization; and [37,38,39], with particle swarm optimization. These approaches have the advantages of flexible behavior in relation to environmental changes and the ability to self-organize. However, as a disadvantage, it can have a very long execution time in cases where the problem is scaled up, making them unsuitable when a solution is needed in real-time [40].

Finally, MRS, with the ability to address constraints such as the dynamic nature and unpredictability of environments, time-consuming tasks, and robot failures, still faces significant challenges.

## 3. Problem Statement and Assumptions

The conceptual task distribution into a group of autonomous robots is not necessarily a pure task allocation. Approaches to MRTA team managing always aim to ensure the accomplishment of a global task through the resolution of sub-tasks. Some concepts can be mistakenly considered as part of the allocation. In this context, these concepts are rigorously defined below.

**Definition** **1.** Assignment *is the method of task allotment to a single worker or staff.*

**Definition** **2.** Scheduling *is the arrangement of tasks or sub-tasks in time, taking into account restrictions, dependencies, and priorities.*

**Definition** **3.** Allocation *assures that scheduled sub-tasks will be assigned to a single worker or group, taking into account the symmetry of workload.*

The task management layer allows the proper characterization of task arrangement in MRS and clarifies that allocation is one piece of the global problem, which will be accomplished only if assignment and scheduling are working together.

The MRTA problem can also be interpreted as a scheduling problem [41]. In this manner, the terms *job* and *task* and the terms *machine* and *robot* are equivalent. The advantage of formalizing MRTA problems as scheduling is that it improves the solution with more specific requirements, resulting in more effective benefits.

The scheduling approach aims to design the distribution of multi-robots taking into account robot restrictions, task constraints, job priority, and resource dependence. Pure task allocation does not address these areas because it is only an instantaneous analysis of robots and groups; the dynamic interaction between tasks is not considered. However, scheduling approaches are always seeking job distribution, managing the job interactions, overseeing the shared resources, and introducing new dynamic features, such as precedence, preemptability, and batching of jobs.

## 4. Warehouse Logistics

**Figure 1:**
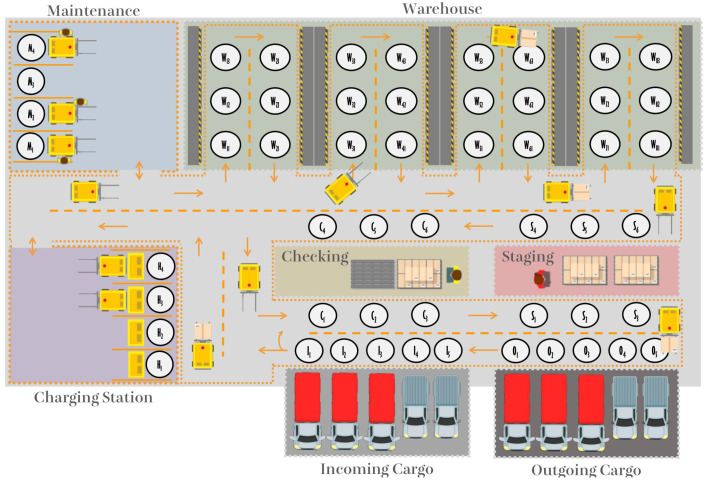
Floor plan of the warehouse logistics with the following sectors: Maintenance, Charging Station, Warehouse, Checking, Staging, Incoming Cargo, and Outgoing Cargo [42] (© 2020 IEEE).

This work is inspired by a real Brazilian warehouse that needs a complete automation process. All sectors of this actual warehouse were reproduced on our warehouse floor plan, shown in Figure 1. In this warehouse, the processes are organized into seven sectors: Incoming Cargo, Checking, Warehouse, Outgoing Cargo, Staging, Maintenance, and Charging Station. In addition, this warehouse has forklifts that can perform various activities such as loading and unloading trucks or shelves. Each white circle, shown in Figure 1, represents a state that can be occupied by only one forklift at a time. Furthermore, transitions between these states, represented by arrows, indicate the direction that forklifts can travel, avoiding collisions.

In this warehouse, when trucks arrive with new goods, they need to park in the Incoming Cargo sector. The forklifts unload the goods, taking them to the Checking sector. In the Checking sector, the conditions of the products are checked, and then they are organized to determine where they will be stored. It is the forklifts that carry the Checking packages to the warehouse. The warehouse is organized into four aisles, according to the type of goods, which can be: automotive items, pharmaceutical items, food items, and miscellaneous items.

**Figure 2:**
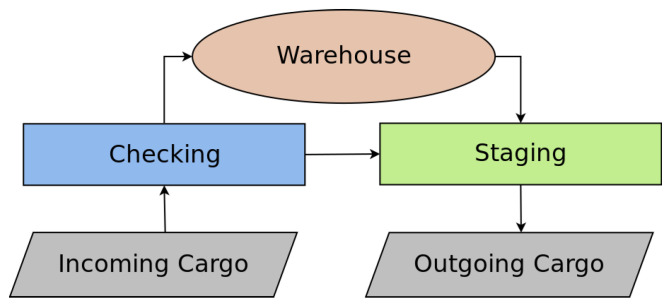
Summary of warehouse logistics process [43] (© 2019 IEEE). Arrows indicate the flow direction of the Incoming Cargo and Outgoing Cargo processes.

On the other hand, to make a delivery to the customer, the goods are first taken from the Warehouse and made available in the Staging sector. In this sector, the goods are packaged for delivery. The next step is to transport these packages by forklift to the Outgoing Cargo sector. Figure 2 presents the interaction between warehouse processes in a simplified manner.

In warehouse logistics, the entry and exit of goods can coincide, setting up a dynamic process. In addition, the number of forklifts that perform tasks changes dynamically according to the number of packages that need to be transported at each entry and exit of goods. Some requests need to be carried out urgently due to consumer contracts or storage conditions such as refrigerated goods.

## 5. Multi-Robot Preemptive Task Scheduling with Fault Recovery (MRPF)

The proposed approach is the expansion of multi-robot management to the scheduling level, allowing task prioritization and fault-tolerance, referred to as MRPF.

**Figure 3:**
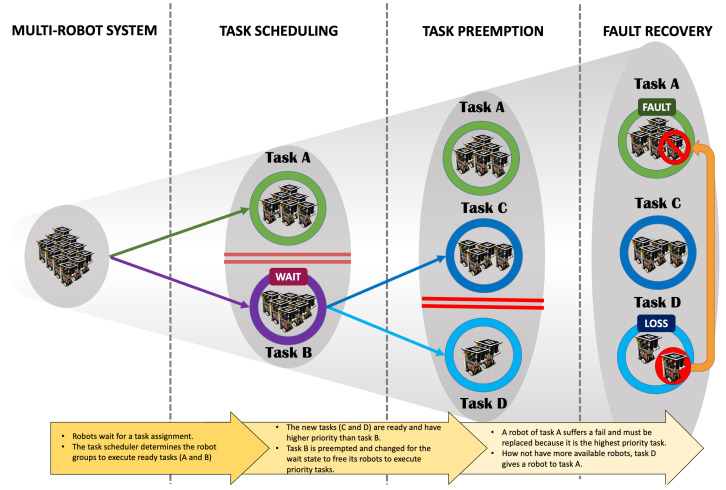
Overview of the MRPF with priority scheduling, task preemption, and fault recovery.

Figure 3 illustrates this approach where two tasks named A and B need to be scheduled. The scheduler then determines which robots will serve them according to their requirements: priority and number of boxes to be transported. However, tasks A and B, when starting their execution, can be interrupted if new processes with a higher priority appear. As in the example in Figure 3, task B had to undergo preemption due to the appearance of tasks C and D with higher priority. Another situation dealt with in the MRPF approach is regarding fault recovery. As an example, Figure 3 presents the situation where, at some point in time, a robot from task A fails and is replaced by an available robot after completing some steps of task D.

MRPF arranges tasks and sub-tasks in time, taking into account restrictions, dependencies, and priorities, updating the arrangement at each changing event. The scheduling cycle is prompted by these events to determine which tasks will be forwarded to the coalition formation, which allocates the tasks to a group of robots to execute.

Tasks are introduced into the warehouse environment with different priorities. The scheduler has the role of determining the execution of each task according to its priority. That is, a low-priority task must be executed after a high-priority task. If a high-priority task comes up and needs to be run at any point, a lower priority task will need to stop running and resume later. The scheduler’s preemption mechanism is activated to stop the execution of the low-priority task, storing its context for future continuation and switching to the execution of the high-priority task.

The interaction between parallel tasks constantly changes the coalitions to prioritize tasks and maintain their requirements. However, the distributed system is susceptible to different fault sources, e.g., low battery and error. In fault occurrence, the robot that failed must communicate with the nearest free robot to initiate a replacement. This mechanism prioritizes the closest robot in accord with its battery state. The replacement can be immediate if there is a free robot or later if it is necessary to wait for a robot to be delocalized to perform the substitution. In any case, the robot that has failed goes to Maintenance.

### 5.1. Characterization of the Tasks

**Figure 4:**
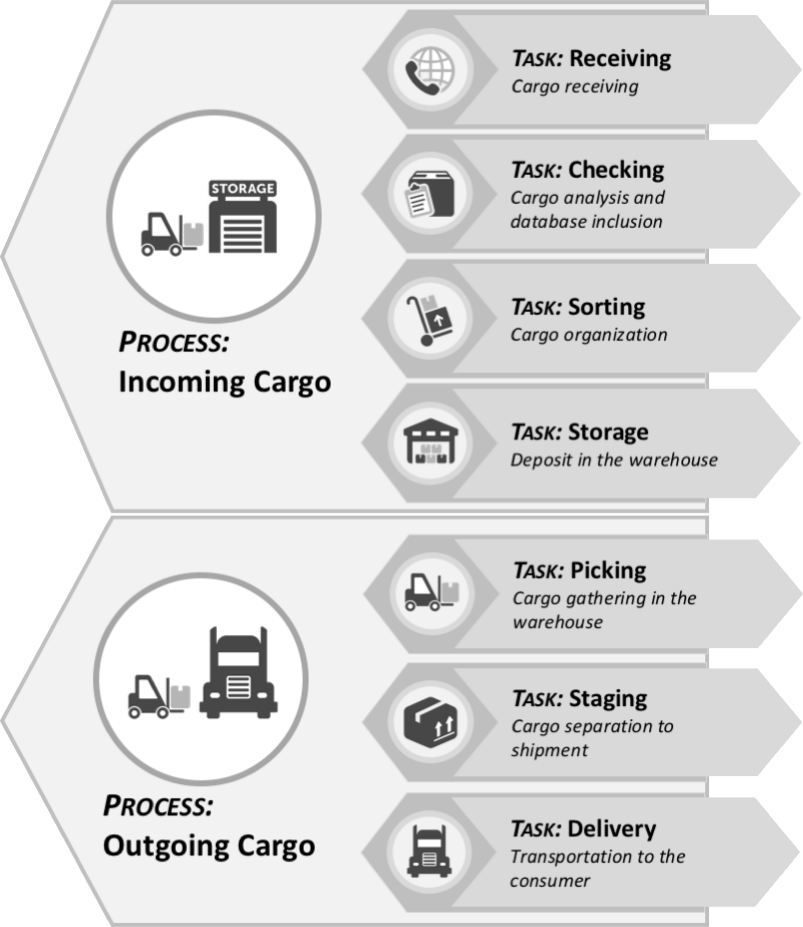
Description of processes, Incoming Cargo and Outgoing Cargo, and their tasks in warehouse logistics [42] (© 2020 IEEE).

Warehouse logistics are responsible for collection, storage, distribution, delivery, and inventory management of items, as illustrated in Figure 1. The logistics are composed of several specific processes and tasks as detailed in Figure 4.

The *Receiving task* is responsible for managing the receipt of goods. When a receipt event occurs, a collection process is started to unload goods in the trucks parked in the *Incoming Cargo* sector. In this case, forklifts must handle goods from *Incoming Cargo* and take them to *Checking*, to be checked and registered in the inventory database, designated as a *Checking task*. After being reviewed, the load must be organized and stored; this is the *Sorting task*. Finally, the *Storage task* addresses storing the cargo in the warehouse.

The *Picking task* begins when there is a customer order to be delivered, which requires withdrawal of the goods from the warehouse and placement in the *Staging* sector. The *Staging task* performs the preparation or packing of goods for delivery. Finally, the cargo dispatching occurs in the *Outgoing Cargo* sector, which is a function of the *Delivery task*.

The logistics scheduler understands the whole operation as the decomposition into processes with high abstraction level (HAL), *Incoming Cargo* and *Outgoing Cargo*. These processes are a sequence of standard tasks (shown in Figure 4). The *Incoming Cargo process* is the sequence of Receiving→Checking→Sorting→Storage. The *Outgoing Cargo process* is the sequence of Picking→Staging→Delivery.

A coalition formation is made for each standard task of HAL processes; that is, groups of different robots can execute these processes. In the execution of tasks, it may be necessary to change a robot because it presents a low battery or an error. A robot will always be replaced by another robot. A coalition can have more than one robot and can also be re-assigned when other robots are closest to the next sub-task to be executed.

### 5.2. Task Scheduling

The MRPF approach maintains a queue of processes, categorized as *Incoming Cargo* or *Outgoing Cargo*. Each process is assigned a priority to determine its importance, which is equally distributed to its tasks. A process is composed of a set of tasks, shown in Figure 4. The *Incoming Cargo* process consists of the following tasks: *Receiving*, *Checking*, *Sorting*, and *Storage*. The *Outgoing Cargo* process consists of the following tasks: *Picking*, *Staging* and *Delivery*. MRPF is designed with four classes of priorities, defined as:

**Priority** **1.** ***Minor***: *These are nonessential processes that do not inhibit the staff functionality or primary purpose of the warehouse. The jobs are executed when possible or by idle workers.*

**Priority** **2.** ***Normal***: *These processes are not Critical or Major, with isolated impact, and may have workarounds (variable staff). They do not have special requirements but must be executed when created. At minimum, one autonomous robot should be made available.*

**Priority** **3.** ***Major***: *This priority is used for processes that are not critical but have a significant impact on warehouse staff. For example, an incoming or outgoing truck with many boxes. This kind of process requires at least two autonomous robots.*

**Priority** **4.** ***Critical***: *These processes must be initiated immediately and completed as soon as possible. This requirement is commonly associated with refrigerated cargo, emergency deliveries, or time-restricted truck stops. At least three autonomous robots are required to execute critical processes.*

The scheduler is designed to perform up to three parallel processes, redirecting resources to new processes with higher priority. The limitation also aims to guarantee the flow of mobile robots in the warehouse, avoiding queues in a crowded environment. Robot distribution is based on priority restrictions. If a given task needs fewer robots than expected, the remaining robots are redistributed, giving preference to the highest priority tasks, or if they have the same priority, to the oldest.

Algorithm 1 briefly presents the operation of the MRPF scheduling process. The scheduling function receives the list of all processes that are being executed or waiting to be served. This function is called within a loop, as the list may undergo new additions to processes to be executed. In line 2, the function *get_priority_process()* selects the process with the highest priority from the list of processes in the *waiting* status. If no processes were waiting, the function ends its processing on line 4. The loop *for* on line 8 checks with which of the three nodes it is possible to execute the process with the highest priority. In line 9, it is verified if the process that is being executed in node *i* has finished its tasks. In this case, the node will have the status *free* (line 10). In line 12, if the node is free, the node receives the process with the highest priority to be executed, updates its status (line 14) to *executing* and the process status also to *executing* (line 15). The *execute()* function is called, which receives a parameter on which node and which process should run on that node. The entire coalition formation and allocation procedure are completed in this function. Finally, in line 17, it is indicated in the *flag_execute* that the process found a node to execute it. In line 19, if the process has not been able to run on that node, the value of the process running with the lowest priority is stored. Finally, on line 26, it is verified that the process has not been executed (*flag_execute == false*) and that its priority is higher than the process running with the lowest priority. If so, the process running with the lowest priority goes to *waiting* status (line 27) and suffers preemption, where its information is saved for later resumption (line 28). Then, the process with the highest priority is executed on line 29.

### 5.3. Task Preemption

A preemption mechanism is adopted to prioritize more important processes, allowing interruption of an active lower priority process. In this case, a lower priority process is stopped and re-enters the process queue. When it is selected again for execution, the process resumes from the point where it stopped.

For example, if an *Incoming Cargo* process has started loading boxes for the *Checking* sector, the process will only be preempted at the end of this step. Upon retake, the process that was preempted will perform the next step, loading into the *Warehouse* sector. The process will only be preempted when the step that is running is finished, not leaving boxes in inappropriate places of the warehouse, which can introduce obstacles to other processes. However, if the robots have not yet started loading, the process will be preempted before it starts.
**Algorithm 1:** MRPF *Scheduling* function. 
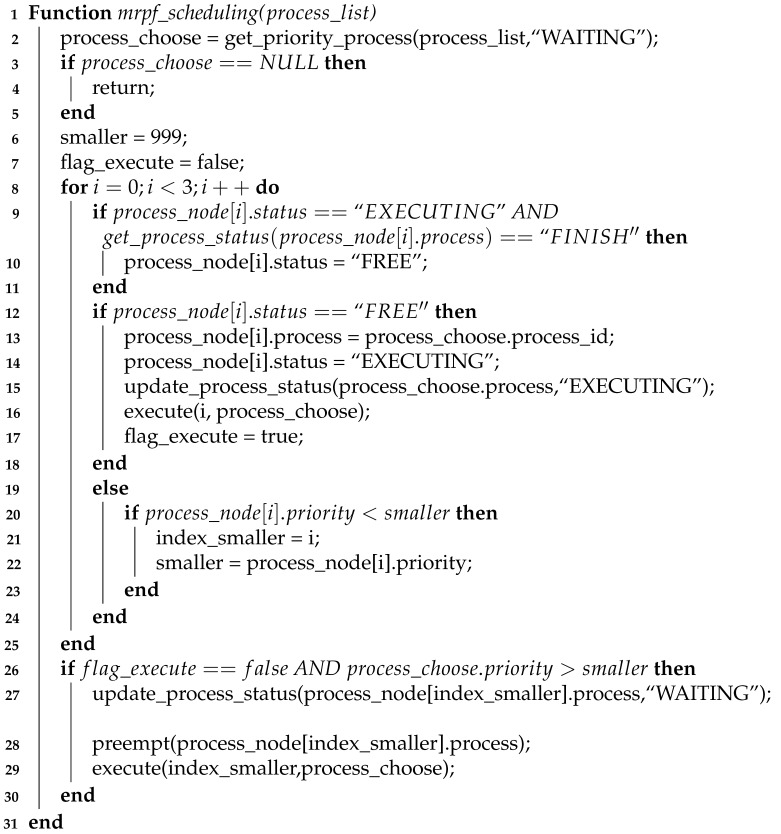


### 5.4. Fault Recovery

During the execution of tasks, robots may fail. In this case, a robot stops executing its task and goes to the *Maintenance* sector. If the failed robot was performing a task, it chooses a new robot to replace it. The criterion of choice for replacement is the closest available robot to the point of failure. The failed robot, if it had cargo, leaves its box where it failed and proceeds to the *Maintenance* sector. The substitute robot proceeds to the fault location and continues the interrupted task. If the replacement robot fails, the procedure is repeated.

If at any point a robot fails and cannot be replaced, the remaining robots move to the current stage of the task. Upon completion, it is checked if any robot can replace the failed robot. At this point, a robot that was performing another task may be free. Again, the nearest robot will be chosen as the replacement.

## 6. Experimental Evaluation of MRPF

**Figure 5:**
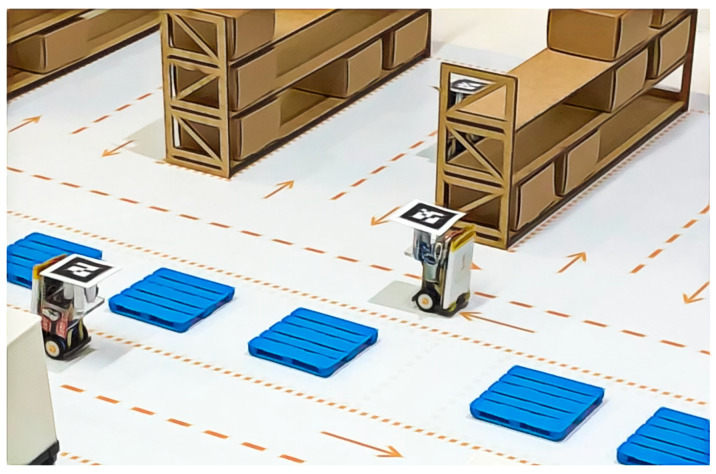
The ARENA, a small-scale warehouse, only with real elements [44] (© 2019 IEEE).

The proposed approach to MRPF is evaluated in a small-scale warehouse called ARENA, which is a physical warehouse that represents a real small warehouse, as shown in Figure 5. The inspiration warehouse is not automated and needs human assistance and control. The goal is to use ARENA with an MRS and augmented reality (AR) to demonstrate an autonomous warehouse system. More details can be obtained at [43].

**Figure 6:**
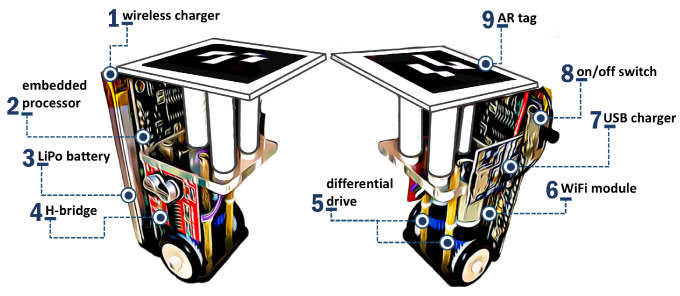
Main components of WsBot: a tiny, low-cost swarm robot [44] (© 2019 IEEE).

Mobile robots replicate the forklift actions of a real warehouse. The tiny robot, called WsBot [44] (Figure 6), was developed for experimentation in small-scale warehouses to evaluate intelligent behavior in smart factories.

The evaluation of the proposed MRPF is planned in three experiments. The first experiment demonstrates an example of priority scheduling. The second experiment illustrates a condition of task preemption. The third experiment analyzes fault detection and recovery.

### 6.1. Experiment 1: Priority Scheduling

**Figure 7:**
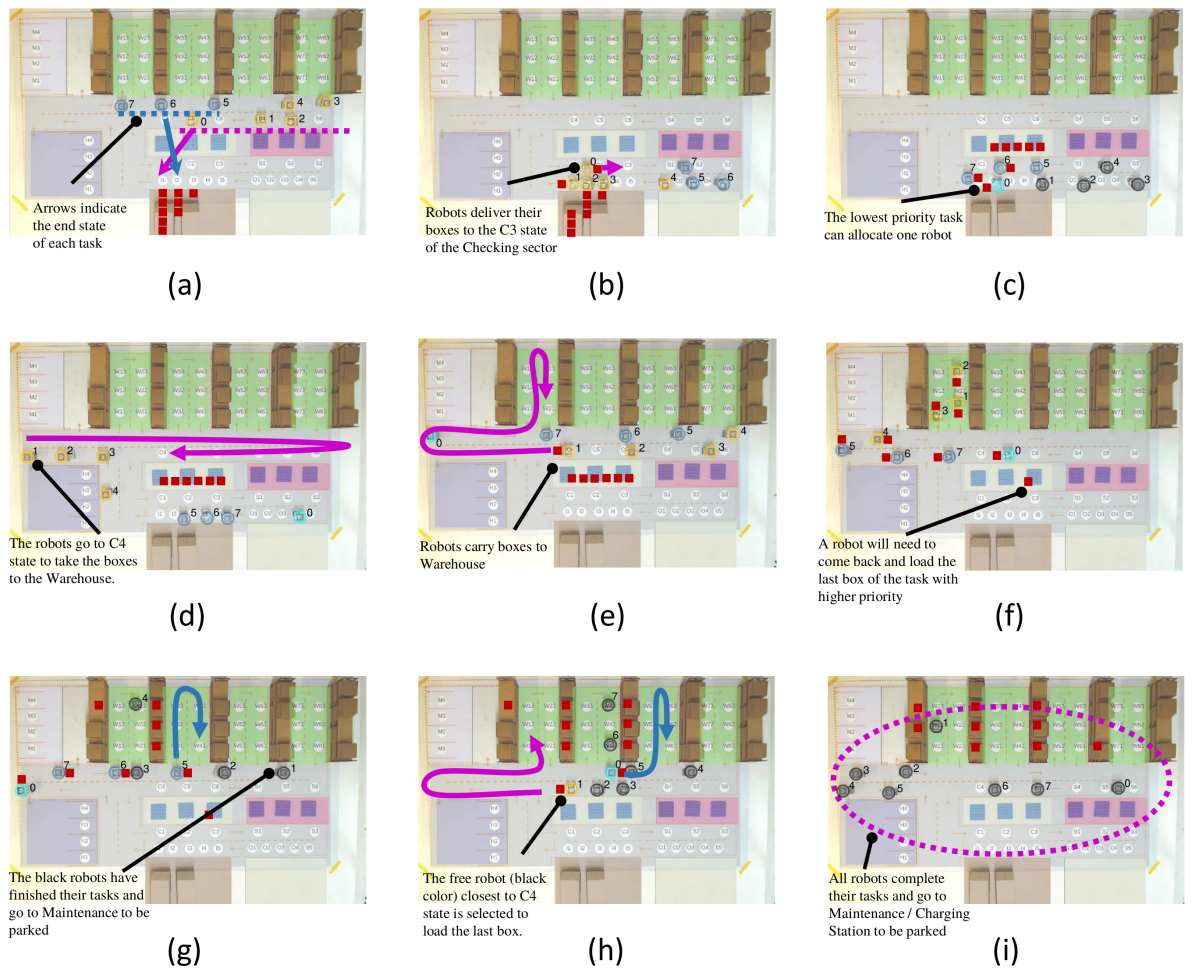
Main scenes from experiment 1: Priority Scheduling. Scene (**a**) being the initial, scene (**i**) the final and scenes (**b**–**h**) the intermediate.

The first experiment is a demonstration of MRPF ability in priority scheduling. In this case, three *Incoming Cargo* processes are performed, as described in Table 1. The three processes are of the *Incoming Cargo* type; that is, they will perform the *Receiving*, *Checking*, *Sorting* and *Storage* tasks. In other words, it aims to transport the boxes that are in the Incoming Cargo sector to the Warehouse. The main scenes of this experiment are shown in Figure 7, and can be seen on YouTube (https://youtu.be/OhSUsAR8kIw, accessed on 19 September 2021).

At the beginning of the experiment, the highest priority process is the only one running. It employs the five robots it needs to load its five boxes, i.e., robots 0–4. The second process receives the remaining three robots, robots 5–7. However, the third process cannot allocate robots immediately and must wait until one of the two processes completes the first step, taking boxes from Incoming Cargo to Checking.

Figure 7a illustrates this moment where the highest priority process robots appear in yellow, and the second-highest priority process robots appear in dark blue. Figure 7b shows the moment when the robots in the first process begin to take boxes from Incoming Cargo to Checking.

A critical moment occurs when the highest priority process robots finish delivering their boxes to the Checking sector (Figure 7c). At this point, robots appear in black to indicate they have been deallocated. The scheduler decides which robots to allocate to the next step of the process, moving the boxes from the Checking sector to the Warehouse. However, because there is a third process waiting for robots, a new distribution is made. In this case, robot 0 (shown in light blue) is allocated to the third process.

Figure 7d shows the moment when the robots that have completed the first process are re-allocated (robots 1–4). The moment when the highest priority process robots begin to retrieve boxes from the Checking sector to take to the Warehouse is shown in Figure 7e. All nine boxes are not visible because they are represented one on top of the other.

In sequence, the highest priority process robots begin delivering the boxes in the Warehouse (robots 1–4), as illustrated in Figure 7f. Next, the robots of the highest priority process are deallocated and appear in black (Figure 7g). In Figure 7h, robot 1 was reassigned to complete the highest priority process, appearing in yellow. It was the robot closest to the Checking sector exit. At this moment, the delivery of boxes by robots 5–7 of the second process is occurring in the second aisle of the Warehouse, and robot 0 of the third process is about to deliver on the third aisle.

The final scene is presented in Figure 7i, with five boxes in the first aisle of the Warehouse, three boxes in the second aisle of the Warehouse, and one box in the third aisle of the Warehouse. The robots turn black to indicate they are free and return to Maintenance or the Charging Station to be parked.

**Figure 8:**
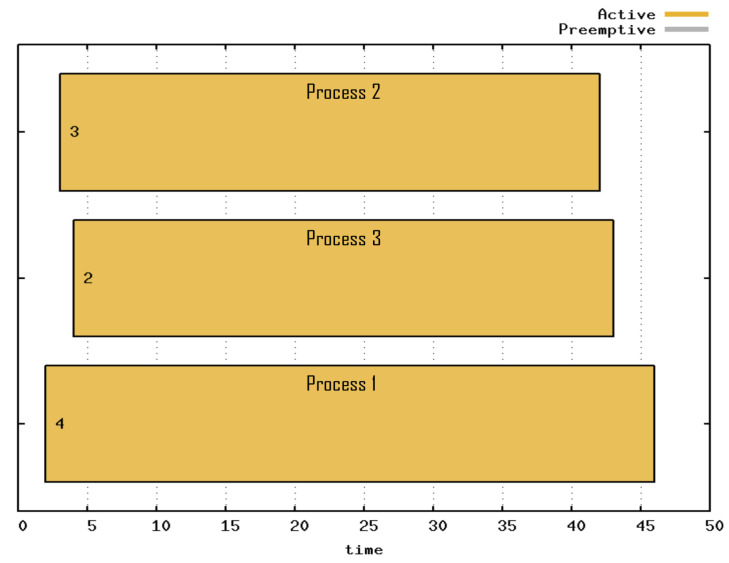
Gantt chart of Priority Scheduling with three processes active throughout the experiment.

The process execution is represented through a Gantt chart (Figure 8), where the number on the chart indicates the priority of each process and the time specified by scheduler cycles, called *ticks*. The yellow color indicates the period in which the processes were running. The process with the highest priority was the first to be attended, but owing to the high number of boxes, it was the last to be completed. The process with the second-highest priority was the second process to be attended and the first process to be completed. The process with the lowest priority was the last to be executed, but because it had only one box, it was the second process to be completed.

### 6.2. Experiment 2: Preemption

**Figure 9:**
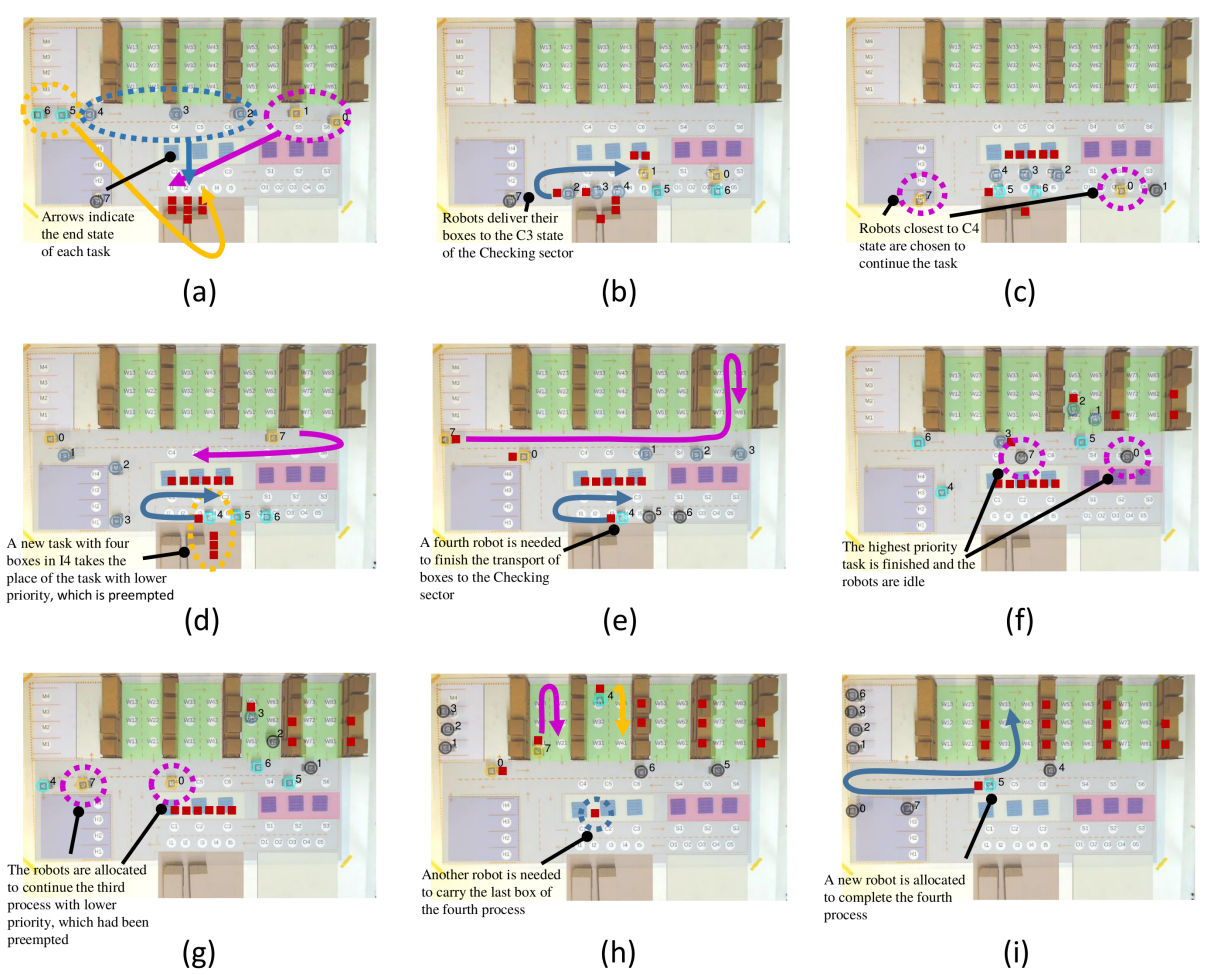
Main scenes from experiment 2: Preemption. Scene (**a**) being the initial, scene (**i**) the final and scenes (**b**–**h**) the intermediate.

The second experiment is an example of preemption when an active process is interrupted in favor of another process with higher priority. The scenario is designed with four *Incoming Cargo* processes, as described in Table 2. The four processes are of the *Incoming Cargo* type; that is, the purpose of these processes is to transport the boxes that are in the Incoming Cargo sector to the Warehouse. The main scenes of this experiment are shown in Figure 9, and can be seen on YouTube (https://youtu.be/RBGzdplPd7w, accessed on 19 September 2021).

The initial scene of this experiment is shown in Figure 9a, where robots 0–1 are allocated to the first process, robots 2–4 are assigned to the second process, and robots 5–6 are allocated to the third process. Figure 9b shows the moment when the robots of the first process deliver their boxes to the Checking sector, and the robots of the second process take their boxes out of Incoming Cargo to transport them to the Checking area.

The robots of the third process (robots 5–6) begin to transport their boxes to the Checking sector, shown in Figure 9c. At this time, robots of the first process were already deallocated (robots 0–1), and new robots were allocated (robots 0 and 7), according to the proximity of the next state of transport, which is the exit of the Checking sector.

In the sequence, the fourth process enters the state I4 (Figure 9d). This process has a higher priority than the third process and considers four boxes that must be delivered to the second aisle of the Warehouse. The state of the third process is saved to be resumed later, when there are no processes running or when it is next by priority. The minimum number of robots that the fourth process could allocate would be two robots, but as the other two processes do not need more robots, the fourth process ends up receiving three robots (robots 4–6). Figure 9e shows the time at which a fourth robot (robot 4) is allocated to load the last box of the fourth process, and robots 5 and 6 are deallocated. It is also the time when the first process robots begin to transport their boxes to the Warehouse (robots 0 and 7).

When the second process robots deliver their boxes in the third aisle of the Warehouse (robots 1–3), as shown in Figure 9f, the first process finishes its execution and deallocates its robots 0 and 7. Thus, Figure 9g shows the moment when robots 0 and 7 are allocated to continue the second stage of the third process.

Next, the third and fourth process robots deliver their boxes into the Warehouse (Figure 9h). However, a box is still missing for the fourth process. The time when a fourth robot is allocated to finish the last transport is shown in Figure 9i (robot 5). Finally, the last scene includes two boxes of the first process in the fourth aisle, three boxes of the second process in the third aisle, two boxes of the third process in the first aisle, and four boxes of the fourth process in the second aisle, as expected.

**Figure 10:**
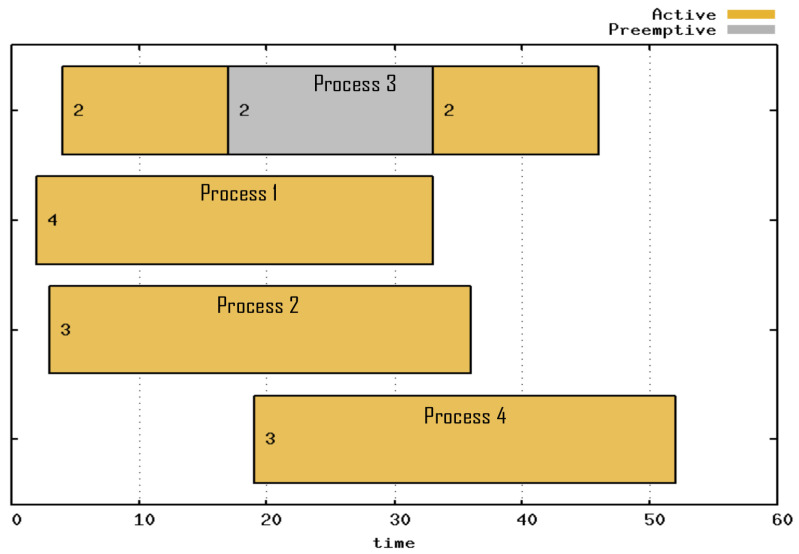
Gantt chart of Preemption, where process 3 was preempted because of process 4, with higher priority.

The Gantt chart of the preemption experiment is presented in Figure 10. The period in which the processes were being executed is indicated in yellow; the period in which a process was preempted is indicated in gray. Processes 1 and 2 were the first to be attended. The process with priority 2 was the third process to be attended and was preempted at time 17. However, the fourth process with priority 3 began to run only in period 19, which was when the third process ended the step that was in progress. At the end of the highest priority process at time 33, the third process resumed and was no longer preempted.

### 6.3. Experiment 3: Fault Recovery

**Figure 11:**
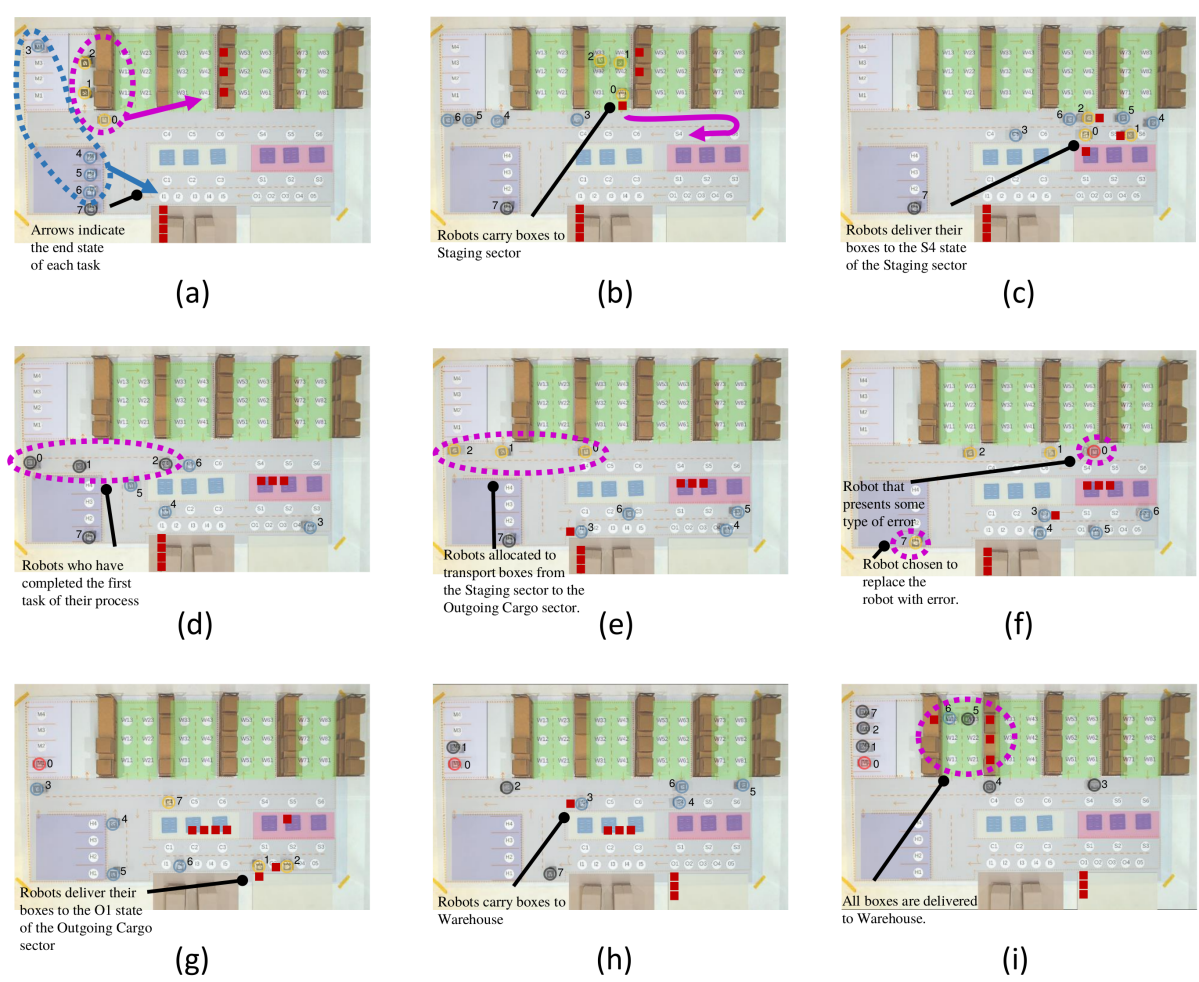
Main scenes from experiment 3: Fault Recovery. Scene (**a**) being the initial, scene (**i**) the final and scenes (**b**–**h**) the intermediate.

The third experiment is an example of fault recovery, where the scheduler reorganizes the coalition to ensure priority execution. In this case, two *Incoming Cargo* processes and one *Outgoing Cargo* process are performed (Table 3). Processes 1 and 3 are of the *Incoming Cargo* type and process 2 is of the *Outgoing Cargo* type. That is, the objective of processes 1 and 3 is to transport the boxes that are in the Incoming Cargo sector to the Warehouse. The objective of process 2 is to transport the boxes from the Warehouse to the Outgoing Cargo sector. The main scenes of this experiment are presented in Figure 11, and can be seen on YouTube (https://youtu.be/kxyLgmSGJnY, accessed on 19 September 2021).

It is assumed that the first process has already been executed, so Figure 11a shows three boxes already in the second aisle. The second and third processes happen simultaneously; the three yellow robots (0–2) are selected for the second process, and the four dark blue robots (3–6) are assigned to the third process. Figure 11b shows the robots of the second process beginning to transport boxes from the Warehouse to the Staging sector. Figure 11c shows the moment the boxes start arriving at Staging.

After delivering the boxes to the Staging sector, the second process robots are deallocated (Figure 11d). A new allocation is made for the second stage of the process, the removal of boxes from the Staging sector and delivery to the Outgoing Cargo sector. The scheduling chooses the robots closest to the exit of the Staging sector, keeping robots 0–2, as shown in Figure 11e. Figure 11e also shows the start of the third process, box loading for the Checking sector.

Robots may experience random errors while performing their processes. In this case, a robot must communicate with the nearest robot for replacement. An example is a fault presented by robot 0 of the second process, shown in red in Figure 11f. Robot 0 is replaced by robot 7, which now appears in yellow. At this point, robot 0 goes to Maintenance for repair, as shown in Figure 11g. Furthermore, note in Figure 11f that robot 3 of the third process begins delivery of its box to the Checking sector.

In Figure 11g, the boxes of the second process begin to arrive at the Outgoing Cargo sector in state O1. Figure 11h shows the three boxes of the second process in the Outgoing Cargo sector and the first box of the third process being transported to the Warehouse. Figure 11i presents the final scene of the simulation, with four boxes referring to the third process, in the first aisle of the Warehouse.

**Figure 12:**
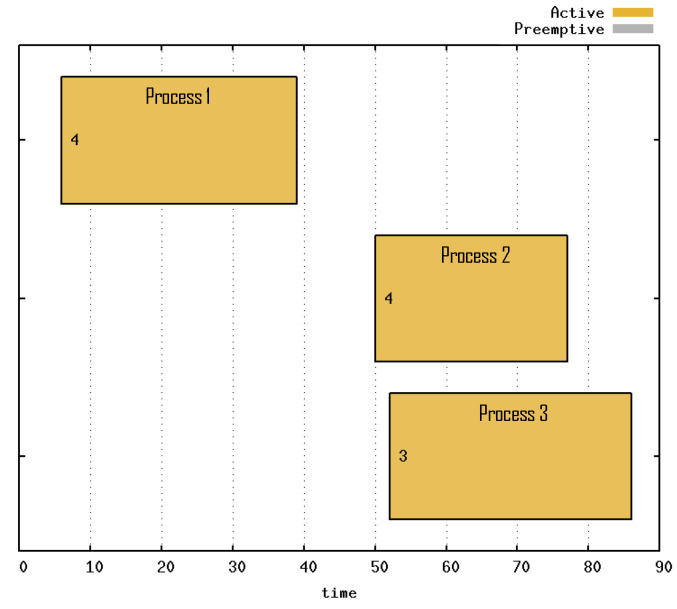
Gantt chart of Fault Recovery for process 2.

The Gantt chart is presented in Figure 12, showing that initially, only the first process was executed. The second and third processes were run simultaneously, and even if a robot of the second process failed and needed to be replaced, it did not hinder progress.

### 6.4. Accuracy and Precision

The accuracy of Multi-Robot Preemptive Task Scheduling with Fault Recovery (MRPF) is evaluated through four distinct experiments that aim to quantify the results. In this case, three *Incoming Cargo* processes and two *Outgoing Cargo* processes are performed, as described in Table 4, where it is assumed that the first process has already been executed.

Processes 2, 3, and 4 are reported to the system simultaneously. Process 5, which has a higher priority than process 3, is informed as soon as process 3 is started. In this case, preemption handling of process 3 is essential to turn process 5. As process 3 starts, the third robot chosen to execute it fails. This situation exemplifies the treatment that should occur in such cases.

The experiments aim to compare the MRPF approach with standard methods, where all cases are evaluated in pure MRTA, MRTA with fault treatment (MRTA + Fault), MRTA with process preemption (MRTA + Preemption), and the proposed MRPF. In addition, four distinct experiments were performed for comparison between these methods.

Table 5 presents the results of the four experiments performed for the MRTA and MRTA + Fault methods, and Table 6 presents the results of the four experiments performed for the MRTA + Preemption and MRPF. Each process has its creation time, in this case, a certain scheduler cycle called *tick* (*Creation tick* column). In addition, the start tick of each process (*Start tick* column) is stored to obtain the delay time to fulfill the process (*Delay tick* column). In the case of MRTA and MRTA + Fault, the *Delay ticks* and *Total delay ticks* column will have the same meaning. However, for MRTA + Preemption and MRPF, the *Total delay ticks* column only considers the active process periods for the summation. The *End tick* column indicates the tick that the process was completed so that the *Elapsed ticks* column can be calculated. For MRTA and MRTA + Fault, *Elapsed ticks*, and *Running ticks* columns have the same meaning. However, for the MRTA + Preemption and MRPF, the sum presented in *Running ticks* considers only the active periods of the processes.

Table 5 shows that for MRTA and the MRTA + Fault, process 5 is created after process 3, and, despite having higher priority than process 3, it only starts its execution after the termination of process 2. Remembering that, it is only possible to execute three processes simultaneously, as seen in Section 5.2. In Table 6, it can be noted for both the MRTA + Preemption and the MRPF that after process 5 is created, process 3 is preempted, giving way to process 5, which has higher priority. The delay ticks of each process emphasize the treatment of preemption. This is more clearly presented in Table 7, where it is noted that the average delay ticks of process 5 in MRTA + Preemption and MRPF is two ticks, while in MRTA and MRTA + Fault it is 7.25 ticks. Consequently, in MRTA + Preemption and MRPF, the average delay ticks of process 3 are longer, as shown in Table 7. However, in MRTA and MRTA + Fault, the delay ticks of process 5 are conditional on the running time of active processes.

Table 5 also shows that the running ticks of process 3 are longer in MRTA scheduler than in MRTA + Fault. This information is also summarized in Table 8. While the average running ticks for process 3 on the MRTA is 41.25 ticks, on the MRTA + Fault it is 33.5 ticks. This is because the third allocated robot to service process 3 fails, and a failure handling mechanism is not provided in the MRTA. Without replacement of the failed robot, the remaining robots need to service the process alone. In the MRTA + Fault, the failed robot is replaced, ensuring that process 3 ends in a shorter time. The same comparison can be made between methods MRTA + Preemption and MRPF. The running ticks of process 3 are longer in the MRTA + Preemption scheduler that has no-fault handling than in the MRPF scheduler.

Table 7 and Table 8 present, respectively, a statistical analysis of the total ticks delay and the total running ticks of the data in Table 5 and Table 6. Table 7 presents information on the maximum (max), minimum (min), population mean value (avg), and population standard deviation (sd) of the performed experiments, referring to the total ticks delay. Table 8 also presents this information; however, it is referring to the total running ticks. This information summarizes the discussion presented for the data in Table 5 and Table 6.

### 6.5. Threats to the MRPF

Some threats are present in the validation of the proposed MRPF approach. The main one is the possibility that all robots fail to complete a task. In that case, the entire multi-robot system would need repair and replacement.

Another situation is that a low-priority task needs to be stopped and saved in case a higher priority task appears to be serviced. When being serviced again, the process with the lowest priority, if it is taking longer than necessary to run, runs the risk of being interrupted again. The aggravation of this situation is related to the importance of the task that has the lowest priority. If the task is from a process with a priority of 1, there is no problem, as this type of process is not essential. However, if it is priority 2 or 3, there will be a problem. In this case, the other processes that need to be included in the system must be rethought as to their urgency and importance, or the less critical process needs to have its priority changed.

## 7. Comparison with Other Approaches

In this section, the MRPF approach is compared to the methods proposed by the authors Hoenig et al. (2018) [45] and Das et al. (2015) [46]. These methods were chosen for comparison because they have code available for evaluation and are easy to adapt to the warehouse scenario.

Hoenig et al. (2018) [45] present some methods based on the *Conflict-Based Search* (CBS) algorithm, named: *Conflict-Based Search—Task Assignment* (CBS-TA), *Enhanced Conflict-Based Search* (ECBS), *Enhanced Conflict-Based Search—Task Assignment* (ECBS-TA), and prioritized planning using *Safe Interval Path Planning* (SIPP). These solutions are designed for collision-free path configuration and task assignment. However, in this paper, the comparison is made only with the CBS-TA and ECBS-TA methods that include assigning tasks. Hoenig et al. (2018) [45] do not address fault recovery.

Das et al. (2015) [46] presents an approach called *Consensus-Based Parallel Auction and Execution* (CBPAE), focused on task allocation in a heterogeneous and autonomous multi-robot system deployed in medical institutions based on auction principles and consensus. However, the CBPAE approach also does not address fault recovery.

**Figure 13:**
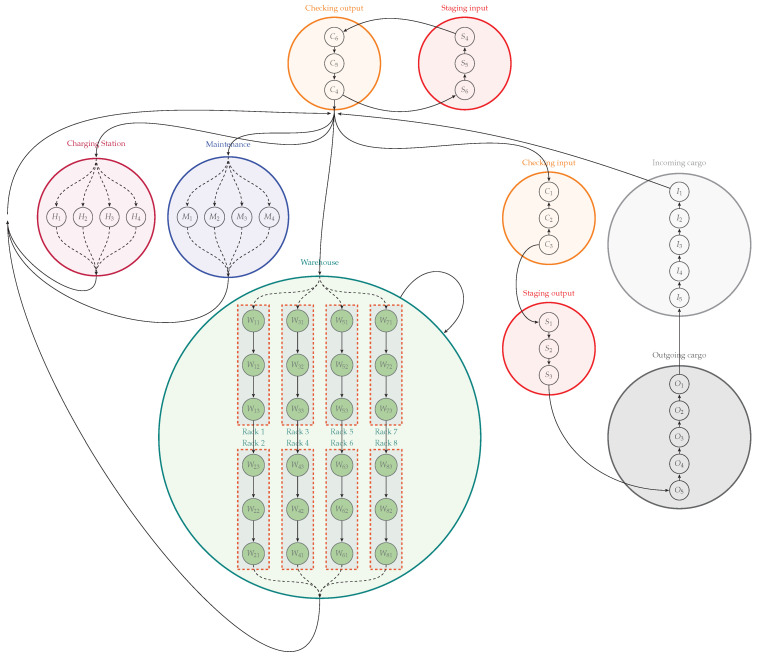
State machine of warehouse logistic process [43] (© 2019 IEEE).

Comparison of the MRPF approach with the works by Hoenig et al. (2018) [45] and Das et al. (2015) [46] considers two objective functions:

- the sum of the travel costs of all robots; that is, the sum of all transitions in the warehouse state machine, as shown in Figure 13. The warehouse state machine displays transitions between states that represent the direction the robots can travel, avoiding collisions between them. Each state can only be occupied by one robot;
- the makespan; that is, the time elapsed between the completion of the first and the last task [47].

ARENA was used for comparison and adapted to the Hoenig et al. [45] and Das et al. [46] approaches. Experiments were carried out with a task involving three boxes for the *Incoming Cargo* and *Outgoing Cargo* processes, presented, respectively, in Table 9 and Table 10.

Table 9 presents the information demonstrating that the MRPF approach presented lower total cost and makespan in all tasks of the *Incoming Cargo* process. This happens because, in the MRPF approach, each robot knows which collision-free graph it must travel, and this graph is one-way.

Table 10 provides the information that the MRPF approach presents the same results as the CBS-TA, ECBS-TA, and CBPAE methods for the picking task, that is, transporting boxes from the *Warehouse* to the entrance of the *Staging* sector. For the next step, which is for the robot to move from the entrance of the *Staging* sector to the exit of the *Staging* sector, the MRPF approach presented the best result both for the total cost and for the makespan. Finally, for the delivery task, which is to transport the boxes from the *Staging* sector to the *Outgoing Cargo* sector, the MRPF approach has the lowest total cost. However, it has the same result as the CBPAE approach of Das et al. (2015) [46] in makespan. For situations where the MRPF approach has better results, the robots know the previously collision-free graph.

The methods were compared in terms of the total cost of robot displacement and process makespan in terms of the Incoming and Outgoing Cargo processes, as shown in Table 11. The MRPF method showed an improvement in relation to the displacement cost of 20.20% compared to CBS-TA, 24.15% compared to ECBS-TA, and 18.33% compared to CBPAE. Regarding makespan, the improvement was 19.63% compared to CBS-TA, 23.21% compared to ECBS-TA, and 17.31% compared to CBPAE.

**Figure 14:**
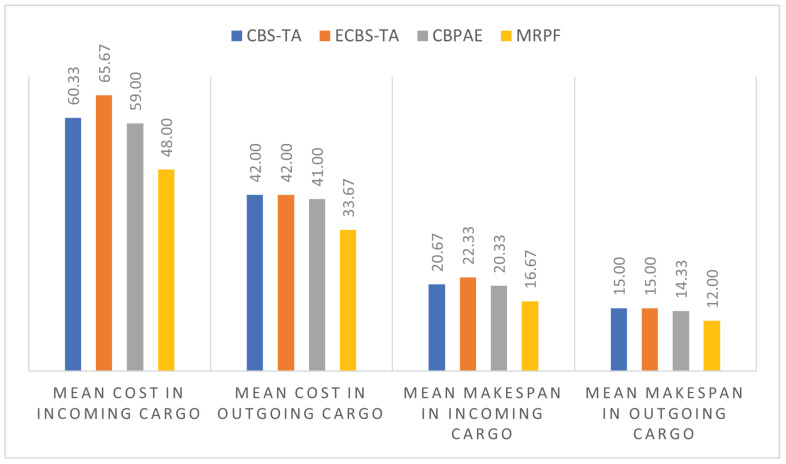
Graphical analysis of the comparison of MRPF with similar methods for Incoming Cargo and Outgoing Cargo processes.

Figure 14 shows the mean cost obtained by comparing the CBS-TA, ECBS-TA, CBPAE, and MRPF methods. In all situations presented, the MRPF method had the lowest mean cost.

## 8. Conclusions

This paper presented MRPF, an approach to designing the distribution of multi-robots, taking into account robot restrictions, task constraints, job priority, and resource dependence. The MRPF approach is focused on distribution during execution, managing the jobs interaction, overseeing the shared resources, and introducing new dynamic features, such as precedence, preemptability, and batching of processes.

Another issue addressed was fault recovery. If a robot fails, it must communicate with the nearest free robot for replacement. The replacement can be immediate if there are robots free, or later if it is necessary to wait for a robot to be delocalized to perform the substitution. In any case, the robot that has failed goes to Maintenance.

A small-scale physical warehouse called ARENA was used to evaluate the proposed MRPF approach. ARENA is a physical warehouse that represents a real small warehouse, with real physical robots called WsBots and using augmented reality to represent the boxes to be transported. The evaluation of the MRPF approach was made through three experiments to prove the benefits of the proposed method. The first experiment demonstrated priority scheduling, with several simultaneous processes run according to priority level. The second experiment explained preemption, i.e., a more critical process can interrupt the execution of a less critical process. Finally, the third experiment studied fault recovery, or how robot faults can be recovered with a new coalition formation. All these features are introduced in MRS to increase efficiency in the industry, improving the production process.

## Figures and Tables

**Table 1:** Description of Experiment 1: Priority Scheduling.

#	Type of Process	Priority	Number of Boxes	Initial State	Warehouse Aisle
Process-1	*Incoming Cargo*	4	5	I1	1
Process-2	*Incoming Cargo*	3	3	I2	2
Process-3	*Incoming Cargo*	2	1	I3	3

**Table 2:** Description of Experiment 2: Preemption.

#	Type of Process	Priority	Number of Boxes	Initial State	Warehouse Aisle
Process-1	*Incoming Cargo*	4	2	I1	4
Process-2	*Incoming Cargo*	3	3	I2	3
Process-3	*Incoming Cargo*	2	2	I3	1
Process-4	*Incoming Cargo*	3	4	I4	2

**Table 3:** Description of Experiment 3: Fault Recovery.

#	Type of Process	Priority	Number of Boxes	Initial State	Warehouse Aisle
Process-1	*Incoming Cargo*	4	3	I1	2
Process-2	*Outgoing Cargo*	4	3	O1	2
Process-3	*Incoming Cargo*	3	4	I1	1

**Table 4:** Description of Experiments.

#	Type of Process	Priority	Number of Boxes	Initial State	Warehouse Aisle
Process-1	*Incoming Cargo*	4	4	I1	1
Process-2	*Outgoing Cargo*	4	1	O1	1
Process-3	*Incoming Cargo*	1	3	I2	2
Process-4	*Incoming Cargo*	3	2	I3	3
Process-5	*Outgoing Cargo*	2	2	O2	1

**Table 5:** MRTA and MRTA + Fault schedulers experiments.

		MRTA Scheduler								MRTA + Fault Scheduler							
Experiment	Process	Creation Tick	Start Tick	Delay Ticks	Total Delay Ticks	End Tick	Status	Elapsed Ticks	Running Ticks	Creation Tick	Start Tick	Delay ticks	Total Delay Ticks	End Tick	Status	Elapsed Ticks	Running Ticks
1	Process-1	2	2	0	0	36	Running	34	34	2	2	0	0	40	Running	38	38
	Process-2	43	43	0	0	57	Running	14	14	51	51	0	0	66	Running	15	15
	Process-3	43	45	2	2	85	Running	40	40	51	53	2	2	87	Running	34	34
	Process-4	44	44	0	0	72	Running	28	28	51	52	1	1	84	Running	32	32
	Process-5	50	57	7	7	81	Running	24	24	58	66	8	8	86	Running	20	20
2	Process-1	2	2	0	0	36	Running	34	34	2	2	0	0	35	Running	33	33
	Process-2	45	45	0	0	59	Running	14	14	44	44	0	0	58	Running	14	14
	Process-3	45	47	2	2	88	Running	41	41	44	46	2	2	79	Running	33	33
	Process-4	45	46	1	1	78	Running	32	32	45	45	0	0	75	Running	30	30
	Process-5	51	59	8	8	92	Running	33	33	51	58	7	7	81	Running	23	23
3	Process-1	2	2	0	0	41	Running	39	39	2	2	0	0	38	Running	36	36
	Process-2	50	50	0	0	65	Running	15	15	48	48	0	0	62	Running	14	14
	Process-3	50	52	2	2	95	Running	43	43	48	50	2	2	85	Running	35	35
	Process-4	51	51	0	0	81	Running	30	30	49	49	0	0	81	Running	32	32
	Process-5	57	65	8	8	83	Running	18	18	55	62	7	7	88	Running	26	26
4	Process-1	2	2	0	0	36	Running	34	34	2	2	0	0	36	Running	34	34
	Process-2	44	44	0	0	57	Running	13	13	45	45	0	0	59	Running	14	14
	Process-3	45	46	1	1	87	Running	41	41	45	47	2	2	79	Running	32	32
	Process-4	45	45	0	0	77	Running	32	32	45	46	1	1	75	Running	29	29
	Process-5	51	57	6	6	80	Running	23	23	52	59	7	7	83	Running	24	24

**Table 6:** MRTA+Preemption and MRPF schedulers experiments.

		MRTA + Preemption Scheduler								MRPF Scheduler							
Experiment	Process	Creation Tick	Start Tick	Delay Ticks	Total Delay Ticks	End Tick	Status	Elapsed Ticks	Running Ticks	Creation Tick	Start Tick	Delay Ticks	Total Delay Ticks	End Tick	Status	Elapsed Ticks	Running Ticks
1	Process-1	2	2	0	0	36	Running	34	34	2	2	0	0	33	Running	31	31
	Process-2	44	44	0	0	58	Running	14	14	40	40	0	0	53	Running	13	13
	Process-3	45	46	1	14	52	Running	6	44	40	42	2	15	47	Running	5	37
			52	-		58	Preemption	6			47	-		53	Preemption	6	
			58	13		96	Running	38			53	13		85	Running	32	
	Process-4	45	45	0	0	74	Running	29	29	40	41	1	1	68	Running	27	27
	Process-5	52	54	2	2	74	Running	20	20	47	49	2	2	68	Running	19	19
2	Process-1	2	2	0	0	36	Running	34	34	2	2	0	0	35	Running	33	33
	Process-2	46	46	0	0	66	Running	20	20	42	42	0	0	61	Running	19	19
	Process-3	46	48	2	22	53	Running	5	41	43	44	1	19	48	Running	4	37
			53	-		66	Preemption	13			48	-		61	Preemption	13	
			66	20		102	Running	36			61	18		94	Running	33	
	Process-4	46	47	1	1	75	Running	28	28	43	43	0	0	69	Running	26	26
	Process-5	53	55	2	2	71	Running	16	16	48	50	2	2	68	Running	18	18
3	Process-1	2	2	0	0	36	Running	34	34	2	2	0	0	33	Running	31	31
	Process-2	44	44	0	0	58	Running	14	14	40	40	0	0	58	Running	18	18
	Process-3	44	46	2	16	51	Running	5	41	41	42	1	18	46	Running	4	32
			51	-		58	Preemption	7			46	-		58	Preemption	12	
			58	14		94	Running	36			58	17		86	Running	28	
	Process-4	45	45	0	0	76	Running	31	31	41	41	0	0	67	Running	26	26
	Process-5	51	53	2	2	76	Running	23	23	46	48	2	2	64	Running	16	16
4	Process-1	2	2	0	0	34	Running	32	32	2	2	0	0	34	Running	32	32
	Process-2	41	41	0	0	59	Running	18	18	41	41	0	0	60	Running	19	19
	Process-3	41	43	2	20	47	Running	4	41	42	43	1	19	48	Running	5	39
			47	-		59	Preemption	12			48	-		60	Preemption	12	
			59	18		96	Running	37			60	18		94	Running	34	
	Process-4	42	42	0	0	66	Running	24	24	42	42	0	0	68	Running	26	26
	Process-5	47	49	2	2	68	Running	19	19	48	50	2	2	65	Running	15	15

**Table 7:** Total ticks delay information.

#	MRTA				MRTA + Fault				MRTA + Preemption				MRPF			
	max	min	avg	sd	max	min	avg	sd	max	min	avg	sd	max	min	avg	sd
**Process-1**	0	0	0.00	0.00	0	0	0.00	0.00	0	0	0.00	0.00	0	0	0.00	0.00
**Process-2**	0	0	0.00	0.00	0	0	0.00	0.00	0	0	0.00	0.00	0	0	0.00	0.00
**Process-3**	2	1	0.75	0.43	2	2	2.00	0.00	22	14	18.00	3.16	19	15	17.75	1.64
**Process-4**	1	0	0.25	0.43	1	0	0.50	0.50	1	0	0.25	0.43	1	0	0.25	0.43
**Process-5**	8	6	7.25	0.83	8	7	7.25	0.43	2	2	2.00	0.00	2	2	2.00	0.00

**Table 8:** Running ticks information.

#	MRTA				MRTA + Fault				MRTA + Preemption				MRPF			
	max	min	avg	sd	max	min	avg	sd	max	min	avg	sd	max	min	avg	sd
**Process-1**	39	34	35.25	2.17	38	33	35.25	1.92	34	32	33.50	0.87	33	31	31.75	0.83
**Process-2**	15	13	14.00	0.71	15	14	14.25	0.43	20	14	16.50	2.60	19	13	17.25	2.49
**Process-3**	43	40	41.25	1.09	35	32	33.50	1.12	44	41	41.75	1.30	39	32	36.25	2.59
**Process-4**	32	28	30.50	1.66	32	29	30.75	1.30	31	24	28.00	2.55	27	26	26.25	0.43
**Process-5**	33	18	24.50	5.41	26	20	23.25	2.17	23	16	19.50	2.50	19	15	17.00	1.58

**Table 9:** Comparison of the MRPF approach with other works for the tasks of the Incoming Cargo process.

	Incoming Cargo/Checking (Entry)				Checking (Entry)/Checking (Exit)				Checking (Exit)/Warehouse			
Method	MRPF	CBS-TA	ECBS-TA	CBPAE	MRPF	CBS-TA	ECBS-TA	CBPAE	MRPF	CBS-TA	ECBS-TA	CBPAE
**Cost of Robot #0**	5	10	13	10	26	27	27	27	17	23	25	22
**Cost of Robot #1**	5	10	13	10	27	28	28	28	16	22	25	21
**Cost of Robot #2**	5	10	12	10	28	29	29	29	15	22	25	20
**Total Cost**	15	30	38	30	81	84	84	84	48	67	75	63
**Makespan**	5	10	13	10	28	29	29	29	17	23	25	22

**Table 10:** Comparison of the MRPF approach with other works for the tasks of the Outgoing Cargo process.

	Warehouse/Staging (Entry)				Staging (Entry)/Staging (Exit)				Staging (Exit)/Outgoing Cargo			
Method	MRPF	CBS-TA	ECBS-TA	CBPAE	MRPF	CBS-TA	ECBS-TA	CBPAE	MRPF	CBS-TA	ECBS-TA	CBPAE
**Cost of Robot #0**	15	15	15	15	10	16	16	16	7	8	8	8
**Cost of Robot #1**	16	16	16	16	10	17	17	17	8	9	9	8
**Cost of Robot #2**	17	17	17	17	11	18	18	18	7	10	10	8
**Total Cost**	48	48	48	48	31	51	51	51	22	27	27	24
**Makespan**	17	17	17	17	11	18	18	18	8	10	10	8

**Table 11:** Summary of comparison of the MRPF with similar methods.

	**COST**			
	**CBS-TA**	**ECBS-TA**	**CBPAE**	**MRPF**
Mean cost in Incoming Cargo	60.33	65.67	59.00	48.00
Mean cost in Outgoing Cargo	42.00	42.00	41.00	33.67
Mean cost of both processes	51.17	53.83	50.00	40.83
**Additional cost to the MRPF**	**20.20%**	**24.15%**	**18.33%**	-
	**MAKESPAN**			
Mean makespan in Incoming Cargo	20.67	22.33	20.33	16.67
Mean makespan in Outgoing Cargo	15.00	15.00	14.33	12.00
Mean makespan of both processes	17.83	18.67	17.33	14.33
**Additional makespan to the MRPF**	**19.63%**	**23.21%**	**17.31%**	-

## Abbreviations

The following abbreviations are used in this manuscript:

ARENA	Augmented Reality to Enhanced Experimentation in Smart Warehouses
CBPAE	Consensus Based Parallel Auction and Execution
CBS	Conflict-Based Search
CBS-TA	Conflict-Based Search—Task Assignment
CPS	Cyber-Physical System
ECBS	Enhanced Conflict-Based Search
ECBS-TA	Enhanced Conflict-Based Search—Task Assignment
MRPF	Multi-Robot Preemptive Task Scheduling with Fault Recovery
MRS	Multi-robot systems
MRTA	Multi-Robot Task Allocation
SIPP	Safe Interval Path Planning
